# Bioactive natural products in COVID-19 therapy

**DOI:** 10.3389/fphar.2022.926507

**Published:** 2022-08-19

**Authors:** Zhonglei Wang, Ning Wang, Liyan Yang, Xian-qing Song

**Affiliations:** ^1^ Key Laboratory of Green Natural Products and Pharmaceutical Intermediates in Colleges and Universities of Shandong Province, School of Chemistry and Chemical Engineering, Qufu Normal University, Qufu, China; ^2^ School of Pharmaceutical Sciences, Tsinghua University, Beijing, China; ^3^ General Surgery Department, Ningbo Fourth Hospital, Xiangshan, China; ^4^ School of Physics and Physical Engineering, Qufu Normal University, Qufu, China

**Keywords:** natural products, COVID-19, SARS-CoV-2, cordycepin, gallinamide A, plitidepsin, telocinobufagin, tylophorine

## Abstract

The devastating COVID-19 pandemic has caused more than six million deaths worldwide during the last 2 years. Effective therapeutic agents are greatly needed, yet promising magic bullets still do not exist. Numerous natural products (cordycepin, gallinamide A, plitidepsin, telocinobufagin, and tylophorine) have been widely studied and play a potential function in treating COVID-19. In this paper, we reviewed published studies (from May 2021 to April 2022) relating closely to bioactive natural products (isolated from medicinal plants, animals products, and marine organisms) in COVID-19 therapy *in vitro* to provide some essential guidance for anti-SARS-CoV-2 drug research and development.

## 1 Introduction

The ongoing coronavirus disease 2019 (COVID-19) pandemic, the sixth public health emergency of international concern, has resulted in 505,035,185 cases and 6,210,719 deaths worldwide during the last 2 years (at the time of writing). (World Health Organization, 2022). The Alpha, Beta, Gamma, and Delta variants of the severe acute respiratory syndrome coronavirus 2 (SARS-CoV-2) responsible for COVID-19 have created recurrent pandemic alerts. (Nasreen et al., 2022). Alarmingly, the novel Omicron (South Africa) variant was firstly confirmed on 24 November 2021. Still, it became the most predominant strain internationally within months because of its increased transmissibility and extensive immune evasion ability. (Scott et al., 2021; Del Rio et al., 2022). Up to now, the devastating Omicron variant has spread to almost all countries. Effective measures, such as vaccines, (Andrews et al., 2022; Chandrashekar et al., 2022) traditional medicine, (Liu et al., 2020; Alam et al., 2021) and small-molecule inhibitors, (Wang and Yang, 2020a; Reis et al., 2022; Sourimant et al., 2022) are greatly needed to reduce human-to-human transmission.

However, promising magic bullets still do not exist. (Kozlov, 2022). As an indispensable resource for promising compounds, natural products have attracted significant attention in countering SARS-CoV-2 infection *via* targeting its main protease (M^pro^, also called 3CL^pro^), (Jin et al., 2020; Mengist et al., 2020) RNA-dependent RNA polymerase (RdRp), (Hillen et al., 2020; Wang et al., 2021a) papain-like protease (PL^pro^), (Yin et al., 2020; Gao et al., 2021) and spike (S) glycoprotein. (Toelzer et al., 2020; Walls et al., 2020). Building on our previously published work, (Wang and Yang, 2020b; Yang and Wang, 2021) we systematically discuss the landmark studies (published between May 2021 and April 2022) relating to bioactive natural products in COVID-19 therapy *in vitro* to support anti-SARS-CoV-2 drugs research and development.

## 2 Promising bioactive natural products in COVID-19 therapy

Bioactive natural products, isolated from medicinal plants, animal products, and marine organisms, are widely studied (in *in vitro*, animal models, and clinical trials) and play an important role in COVID-19 therapy. (Wei et al., 2020; Sahoo et al., 2021; Alqathama et al., 2022). Natural products are still considered one of the most positive and practical approaches to defeating the ongoing pandemic.

**FIGURE 1:**
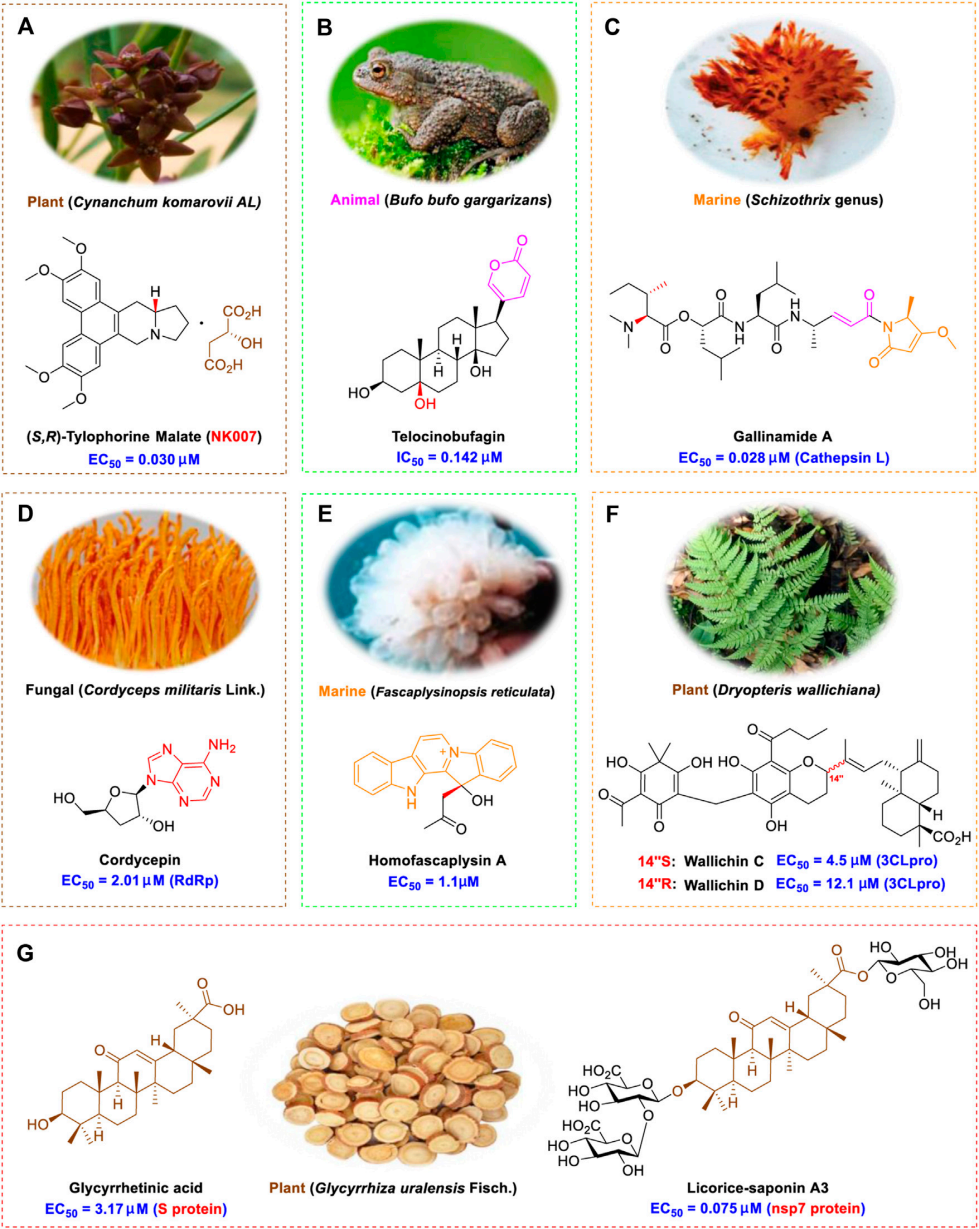
Promising natural products in COVID-19 therapy. **(A)** Tylophorine can be isolated from the medicinal plant *Cynanchum komarovii* AL. **(B)** Telocinobufagin can be isolated from the traditional medicinal animal toad *Bufo gargarizans*. **(C)** Gallinamide A can be isolated from the marine cyanobacteria *Schizothrix* genus. **(D)** Cordycepin can be isolated from the traditional medicine *Cordyceps militaris* Link. **(E)** Homofascaplysin A can be isolated from the marine sponge *Fascaplysinopsis reticulata*. **(F)** Wallichins C and D can be isolated from the medicinal fern *Dryopteris wallichiana*. **(G)** Licorice-saponin A3 and glycyrrhetinic acid can be isolated from the medicinal plant *Glycyrrhiza uralensis* Fisch.

Tylophorine, a remarkable tylophora alkaloid, is an active pharmaceutical ingredient of the medicinal plant *Cynanchum komarovii* AL (Figure 1A) (An et al., 2001). NK007(*S,R*), a racemate of tylophorine malate, was prepared from *S*-tylophorine to improve its poor solubility. (Wang et al., 2010). NK007(*S,R*) displays significant inhibitory activity against SARS-CoV-2 at a half maximal effective concentration (EC_50_) of 0.030 μM in Vero cells, with an excellent selectivity profile (selectivity index, [SI] = 868). (Wang et al., 2021b). Hossain et al. (2022) found that tylophorine showed binding affinity (−8.5 kcal/mol) against abelson murine leukemia viral oncogene homolog one protein. Additionally, NK007(*S,R*) exhibits excellent *in vivo* antiviral efficacy in the COVID-19 golden hamster rat model by significantly reducing viral loads in the lungs. NK007(*S,R*) could protect against lung injury by decreasing lung inflammation with a dose of 5 mg/kg. (Wang et al., 2021b). Briefly, the abovementioned evidence has highlighted the superior activity of NK007(S,R) against SARS-CoV-2 infection in *in vitro* and in the rat model. (Wang et al., 2021b). Numerous natural product-based nanomedicines have been sprung up during the past several decades in the field of medicinal chemistry, providing a valuable reference for anti-COVID-19 therapeutics. (Sharma et al., 2021). To evaluate the potential of the candidate NK007(S,R), Wang et al. (Wang et al., 2021b) prepared self-assembled poly (ethylene glycol)–poly (lactide-co-glycolide) nanoparticles, NP-NK007 and LP-NK007. The optimized NP-NK007 exhibited small particle size (145.8 nm), high NK007(S,R) loading (13.10%), maximized encapsulation efficiency (87.47%), and sustained release (66.51% in 48 h). The optimal lung-targeted liposome LP-NK007 exhibited smaller particle size (75 nm), higher drug loading (36.7%), and excellent encapsulation efficiency (62.4%). Subsequent experiments implied that the nanoparticles NP-NK007 and LP-NK007 are effective SARS-CoV-2 inhibitors with higher EC_50_ values of 0.007 and 0.014 μM, respectively, because they improve the accumulation and delivered efficiency of NK007(S,R) in the lung. (Wang et al., 2021b). Collectively, NK007(S,R) NPs could provide a workable strategy for overcoming the lack of COVID-19-targeting treatment. Theoretically, more validation studies *in vivo* are needed to systematically assess the anti-SARS-CoV-2 potential of NK007(S,R)-based nanoparticles.

Venenum Bufonis (Chinese name: ChanSu), a well-known secretion of a traditional medicine animal (toad *Bufo bufo gargarizans*), is commonly used in China to treat various diseases, including heart failure, infections, toothaches, and cancers. (Tian et al., 2017; Shen et al., 2022). For example, Huachansu injection, a valuable anticancer agent, has been used in tumour treatment in China for more than 30 years. (Wu et al., 2022a). ChanSu’s main active constituents are bufadienolides that have an unusual 2-pyrone ring, which contributes to their pharmacological activities *via* inhibiting Na^+^/K^+^ ATPase. (Prassas and Diamandis, 2008). Recently, Jin et al. (2021) demonstrated that six bufadienolides (bufalin, bufotalin, cinobufagin, cinobufotalin, resibufogenin, and telocinobufagin) have potent broad-spectrum antiviral activities *in vitro* (Figure 1B). Experiments showed that bufalin could inhibit virus replication in the nanomolar range, including MERS-CoV at a half-maximal inhibitory concentration (IC_50_) of 0.018 μM, SARS-CoV at an IC_50_ of 0.016 μM, and SARS-CoV-2 at an IC_50_ of 0.019 μM; cinobufagin can inhibit MERS-CoV, SARS-CoV, and SARS-CoV-2 replication at IC_50_ values of 0.017, 0.060, and 0.072 μM; telocinobufagin can inhibit MERS-CoV, SARS-CoV and SARS-CoV-2 replication with IC_50_ values of 0.027, 0.071, and 0.142 μM; bufotalin, cinobufotalin and resibufogenin can inhibit the MERS-CoV, SARS-CoV and SARS-CoV-2 replication *in vitro* with high IC_50_ values (0.027–1.612 µM). (Jin et al., 2021). This study showed that the unusual 2-pyrone ring in bufadienolides plays an essential role in inhibiting SARS-CoV-2 replication. Subsequent dose toxicity studies (10 mg/kg/day, 5 days) revealed that bufalin and cinobufagin have strong toxicity in the mouse model, while the pharmacokinetic model predicts that telocinobufagin has lower toxicity, better metabolic stability, excellent oral bioavailability, and proper anti-SARS-CoV-2 activity. (Jin et al., 2021). Taken together, telocinobufagin might be a more promising broad‐spectrum inhibitor among the bufadienolides, and thus worthy of multifaceted properties investigation from *in vitro* studies to clinical practice.

Gallinamide A, possessing an α,β-unsaturated imide moiety, is a novel linear depsipeptide first isolated in 2008 from the marine cyanobacteria *Schizothrix* genus and *Symploca* sp. with critical pharmacological effects (Figure 1C) (Linington et al., 2009; Taori et al., 2009). Gallinamide A is a highly selective covalent inhibitor targeting human cathepsin L-like cysteine proteases, which is a promising drug target. (Barbosa Da Silva et al., 2022). Gerwick’s group showed that gallinamide A had a 28- to 320-fold higher affinity and selectivity towards cathepsin L than cathepsin V or B. (Miller et al., 2014). *In vitro*, gallinamide A demonstrates significant bioactivity against *Trypanosoma cruzi* at an IC_50_ of 0.005 μM by irreversible Michael addition. (Miller et al., 2014). It has been reported that gallinamide A can decrease viral load in VeroE6 cells with an IC_90_ of 0.088 µM and inhibit SARS-CoV-2 cathepsin L-mediated endosomal entry with an EC_50_ value of 0.028 µM in a dose-dependent manner. (Ashhurst et al., 2022). Angiotensin-converting enzyme 2 (ACE2) and transmembrane serine protease 2 (TMPRSS2) are two essential host determinants for SARS-CoV-2 infection and pathogenesis *in vivo*. (Hoffmann et al., 2020). Specifically, the S glycoprotein helps the virus enter inside the host cell *via* cellular receptor ACE2 binding; then TMPRSS2 helps SARS-CoV-2 contents fuse and release into the host cell cytosol *via* enzymatical activation of the S glycoprotein. (Liu et al., 2022). Based on combination drug therapies, Payne et al. (Ashhurst et al., 2022) recently demonstrated that the combined use of the cathepsin L inhibitor gallinamide A and the TMPRSS2 protease inhibitor nafamostat mesylate exerts a synergistic inhibitory effect in HEK-ACE2-TMPRSS2 cells *via* inhibiting multiple routes of SARS-CoV-2 entry. Taking gallinamide A as the lead, Payne et al. (Ashhurst et al., 2022) further explored and synthesized 32 analogues for the assessment of SARS-CoV-2 cathepsin L inhibitory activities; the study revealed two lead analogues of gallinamide A with EC_50_ values in the nanomolar range. Taken together, gallinamide A is a highly selective SARS-CoV-2 cathepsin L inhibitor, thus worthy of further investigation *via* combination therapies and lead optimization.

Natural products with broad‐spectrum bioactivities and multi-organ protection are an essential class of anti‐SARS‐CoV‐2 agents that play vital roles in COVID-19 therapy. (Wang and Yang, 2021). RdRp could regulate viral replication through catalyzing the RNA template–dependent development of phosphodiester bonds. (Wang et al., 2021a). The adenosine analogue cordycepin (3′-deoxyadenosine) is a unique fungal product isolated from the traditional medicine fungi *Cordyceps militaris* (Cunningham et al., 1950) and *Ophiocordyceps sinensis* (Figure 1D) (Zhou et al., 2008). Interestingly, cordycepin is known to have broad‐spectrum pharmacological properties against several diseases (e.g., virulent RNA viruses) and multi-organ protective effects (e.g. acute lung injury). Specifically, cordycepin is a promising therapeutic against several viruses *in vitro*, including dengue virus, (Panya et al., 2021) Epstein-Barr virus, (Choi et al., 2019) and hepatitis C virus. (Ueda et al., 2014). Because of its close structural similarity to the cellular nucleoside adenosine (except for the absence of a hydroxyl group at the 3′-position of the five-membered ring), cordycepin is a possible potent anti-SARS-CoV-2 agent. Rabie et al. (Rabie, 2022) showed that cordycepin could inhibit SARS-CoV-2 replication in Vero E6 cells with an EC_50_ value of 2.01 µM and without observable cytotoxicity (SI > 49.8) in a time-dependent manner. It is worth noting that cordycepin is a long-acting antiviral for SARS-CoV-2 prevention with high metabolic stability, reaching maximal anti-SARS-CoV-2 potency within 1.5–2.0 days of treatment. (Rabie, 2022). With respect to the activation mechanism, cordycepin is rapidly converted *in vivo* to its mono-, di-, and triphosphate forms; then, the active form cordycepin triphosphate can serve as a substrate for the RNA-dependent RNA polymerase (RdRp) to terminate the synthesis of viral RNA sequences. (Rabie, 2022). Bibi et al. (2022) revealed that two pivotal amino acid residues (Asp760 and Asp761) play critical roles in the binding of cordycepin with RdRp. Notably, SARS-CoV-2 infection—even in mild cases—can increase the long-term risk of a broad range of cardiovascular and cerebrovascular complications in COVID-19 patients. (Wang and Yang, 2022c). In terms of organ protection, cordycepin has unique advantages. For example, cordycepin plays a key role in long-term neuroprotection for traumatic brain injury (through inhibiting neutrophil infiltration and preserving neuroinflammation), (Wei et al., 2021) protecting diabetic hearts from ischemia/reperfusion injury (*via* up-regulating AMPK/Mfn2-dependent mitochondrial fusion and expression), (Yu et al., 2021) and ameliorating cerebral ischemic damage (*via* improving the memory ability, up-regulating the level of adenosine A1 receptors, and reducing dendritic morphology scathing). (Chen et al., 2021). Thus, cordycepin has its advantages in organ protection and broad-spectrum antiviral activities. Further study is still needed, however, to evaluate its antiviral potency *in vitro*.

The marine environment is a valuable source of structurally unique natural products with diverse bioactivity targeted at life-threatening diseases, including the emerging COVID-19. (Panggabean et al., 2022; Pokharkar et al., 2022; Zhang et al., 2022). Homofascaplysin A, isolated from the marine sponge *Fascaplysinopsis reticulata* (Figure 1E), is a well-established β-carboline alkaloid reported to exhibit promising activity against many viruses, including hepatitis C virus, (Ishida et al., 2001) human coronavirus NL63, (Tsai et al., 2020) and dengue virus. (Quintana et al., 2016). Kubanek et al. (Chhetri et al., 2022) revealed that homofascaplysin A can inhibit SARS-CoV-2 replication in Calu-3 cells at an EC_50_ value of 1.1 µM with relatively slight cytotoxicity (SI ∼4.55). Additionally, Kubanek et al. (Chhetri et al., 2022) found that the viral load was substantially reduced (by >90%) for infections in harvested SARS-CoV-2 RNA after administration of 2.8 μM of homofascaplysin A. Therefore, homofascaplysin A could be used as a unique lead compound for the rapid screening of novel analogues with promising anti-SARS-CoV-2 activity and minimal cytotoxicity.

Chirality is a critical attribute of natural products. (Wang, 2019). Wallichin C and wallichin D, isolated from the medicinal fern *Dryopteris wallichiana* (Figure 1F), exhibit potent anti-SARS-CoV-2 activities in Vero-E6 cells at EC_50_ values of 4.5 and 12.1 μM, respectively. (Socolsky et al., 2012; Hou et al., 2022). The corresponding SI values of wallichins C and D were >35 and >11. (Hou et al., 2022). Furthermore, phloroglucinol-terpenoids wallichins C and D exhibit potent inhibitory activities in SARS-CoV-2-infected Calu-3 cells at EC_50_ values of 20.2 and 30.0 μM, with moderate cytotoxicity (SI values were 4.88 and 2.14 μM, respectively). (Hou et al., 2022). Notably, both wallichins C and D have the same core structure except for the chirality at C-14 position. The study has demonstrated that the slight differences in the chirality at C-14″ S (wallichin C) or C-14″ R (wallichin D) account for differences in their antiviral activities. (Hou et al., 2022). As for the activation mechanism, Zhou et al. (Hou et al., 2022) unambiguously showed that wallichins C and D have higher selectivity and stronger interaction toward the 3CL^pro^ with K_d_ values of 12.0–16.6 μM, while not active against the TMPRSS2, spike glycoprotein, and ACE2 proteins. Taken together, wallichin C might be the more promising 3CL^pro^ inhibitor, thus worthy of further investigation.

Among pharmacological interventions, traditional medicine plays a positive role in the prevention and treatment of the COVID-19 pandemic. (Lyu et al., 2021; Zhan et al., 2022). For example, the Qingfei Paidu decoction has shown amazing clinical efficacy in treating COVID-19 patients. (Li Y. et al., 2021). It is crucial to support scientific foundations for the clinical use of Chinese herbal medicine by exploring the underlying molecular mechanisms. (Cui et al., 2021; De Jin et al., 2021). Ye and co-workers (Yi et al., 2022) recently indicated that licorice-saponin A3 and its genine aglycone glycyrrhetinic acid, famous triterpenoids that could be isolated from the most frequently used medicinal plant *Glycyrrhiza uralensis* Fisch. (Figure 1G), show a remarkably different inhibitory potency against SARS-CoV-2 infection in Vero E6 cells at EC_50_ values of 0.075 μM (targeting SARS-CoV-2 nsp7 protein) and 3.17 μM (targeting the S protein receptor-binding domain [RBD]), respectively in a dose-dependent manner. Interestingly, licorice-saponin A3 and glycyrrhetinic acid were effective in inhibiting the SARS-CoV-2 spike RBD activities, with similar IC_50_ values of 8.3 and 10.9 μM, respectively. (Yi et al., 2022). To elucidate the remarkable difference between S-RBD inhibitory effects and their antiviral activities, the underlying molecular mechanisms were further explored by Ye and co-workers. Based on molecular docking analysis of licorice-saponin A3 with nsp7 (PDB ID:7JIT), Yi et al. (2022) propose that nsp7 is another vital target for licorice-saponin A3 *via* seven hydrogen bond interactions (binding energy −8.7 kcal/mol). Qingfei Paidu decoction extracted from 21 types of traditional Chinese medicines (including *Glycyrrhiza uralensis* Fisch.) could effectively treat COVID-19, highlighting an important contributor to the active components (such as licorice-saponin A3, glycyrrhetinic acid, and so on) in herbal medicine treatment. (Wu et al., 2022b). Importantly, the results provided valuable data on the “multi-components, multiple-pathways, and multi-targets” feature of traditional herbal medicine.

Glycosylation is an important structural modification that increases water solubility, enhances pharmacological activity, and improves the bioavailability of natural products. 11) In fact, GA mainly exists in the form of functional glycosides in licorice. At present, more than 43 saponins have been identified in licorice, many of which are glycosylated derivatives of GA. 12) These glycosylated derivatives have different sugar numbers and types and display various pharmacological activities.

## 3 Other promising natural products for treating SARS-CoV-2 infection

Innovative drug development is an arduous process; bioactive natural products greatly expedite the development of antiviral drugs. (Abdelmohsen et al., 2017). In addition to the abovementioned agents, numerous other natural products (Table 1) have exhibited highly efficacious anti-SARS-CoV-2 activities *in vitro* and clinical practice. For example, plitidepsin (Aplidin^®^), a eukaryotic translation elongation factor 1A (eEF1A) inhibitor of marine origin, was initially approved to treat multiple myeloma. (Rodon et al., 2021). Sachse et al. (2022) showed that plitidepsin is highly effective at inhibiting SARS‐CoV‐2 replication in a dose-dependent manner in Vero E6 cells at IC_50_ values of 0.0052 μM for D614G variants, 0.0039 μM for Delta variants, and 0.0043 μM for Omicron variants. Furthermore, White et al. (2021) showed that plitidepsin can inhibit SARS-CoV-2 replication in Vero E6 cells, hACE2-293T cells and pneumocyte-like cells at IC_50_ values of 0.00070, 0.00073, and 0.0016 μM, respectively, *via* targeting the host protein eEF1A. Notably, Guisado-Vasco et al. (2022) showed that plitidepsin is well-tolerated in humans and can lower viral load in SARS-CoV-2-infected chronic lymphocytic leukemia patients. Clinical trials of plitidepsin have been registered (NCT04382066 and NCT05121740) and will be reported shortly. Further study is still needed to evaluate its anti-SARS-CoV-2 potency *in vivo* and *in vitro*.

**TABLE 1:** Other promising natural products for treating SARS-CoV-2 infection *in vitro*.

No.	Name	Structure	EC_50_ or IC_50_ (μM)	Strain	Refs
1	Aloin A	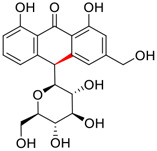	15.68	Vero E6 cells	Lewis et al. (2022)
2	Aloin B	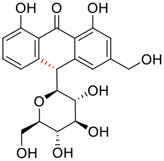	17.51	Vero E6 cells	Lewis et al. (2022)
3	Andrographolide	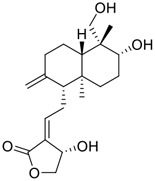	0.034	Calu-3 cells	Sa-Ngiamsuntorn et al. (2021), Schulte et al. (2022)
4	Aspulvinone D	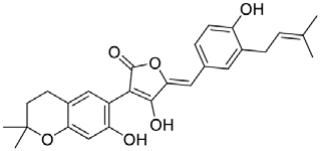	10.3	J774A.1 cells	Liang et al. (2022)
5	Aspulvinone M	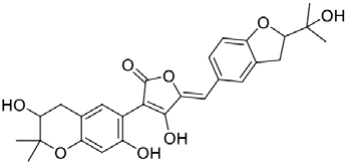	9.4	J774A.1 cells	Liang et al. (2022)
6	Aspulvinone R	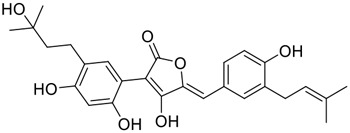	7.7	J774A.1 cells	Liang et al. (2022)
7	(+)-Aureol	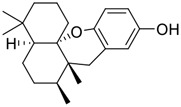	4.00	Calu-3 cells	Chhetri et al. (2022)
8	Baicalein	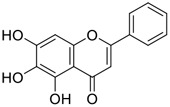	1.11	*E. coli* BL21 cells	Wu et al., 2022a, Xiao et al. (2021)
9	Baicalin	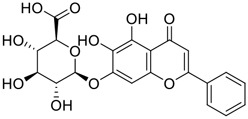	8.8	Vero E6 cells	Ngwe Tun et al. (2022)
10	Bromophycolide A	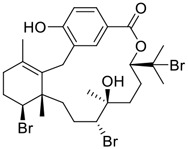	6.90	Calu-3 cells	Chhetri et al. (2022)
11	Bufotalin	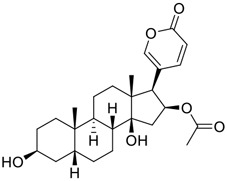	0.072	Vero E6 cells	Jin et al. (2021)
12	Cannabidiol	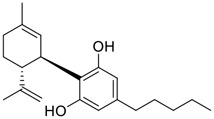	1.24	A549-ACE2 cells	Corpetti et al. (2021), Nguyen et al. (2022)
13	Cannabidiolic acid	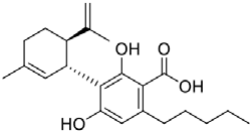	24 μg/mL	Vero E6 cells	van Breemen et al. (2022)
14	Cannabigerolic acid	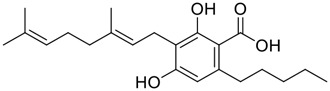	37 μg/mL	Vero E6 cells	van Breemen et al. (2022)
15	Chebulagic acid	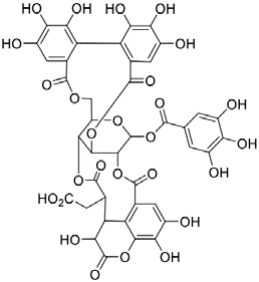	9.76	Vero E6 cells	Du et al. (2021)
16	Cinobufagin	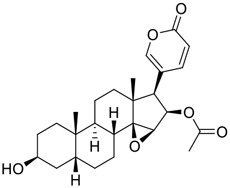	0.072	Vero E6 cells	Corpetti et al. (2021), Nguyen et al. (2022)
17	Cinobufotalin	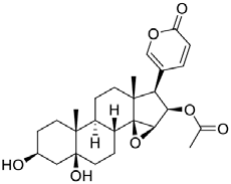	0.399	Vero E6 cells	Jin et al. (2021)
18	Corilagin	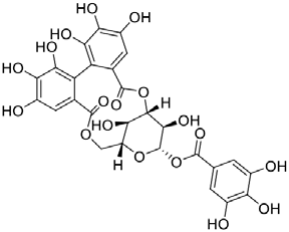	24.9	HEK293 cells	Yang et al. (2021a)
19	Curcumin	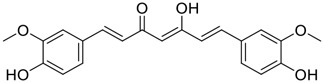	11.9	Vero E6 cells	Bahun et al. (2022)
20	Cyclopeniol	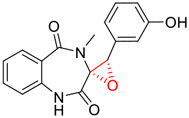	0.39	RAW264.7 cells	Thissera et al. (2021)
21	Cyclopeptin	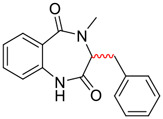	0.40	RAW264.7 cells	Thissera et al. (2021)
22	Dehydrocyclopeptin	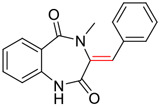	0.89	RAW264.7 cells	Thissera et al. (2021)
23	Dieckol	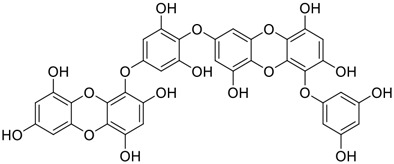	4.50	Vero E6 cells	Yan et al. (2021)
24	Digitoxin	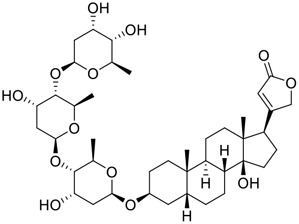	0.059	Vero E6 cells	Caohuy et al. (2021), Jin et al. (2021), Caohuy et al. (2022)
25	Dihydromyricetin	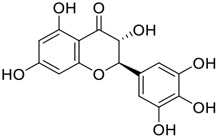	1.14	Vero E6 cells	Su et al. (2021), Xiao et al. (2021)
26	Dihydrotanshinone I	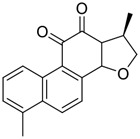	8.14	Vero E6 cells	Ma and Wang, (2022)
27	Dithymoquinone	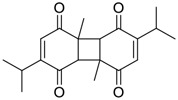	0.275 μg/mL	Vero E6 cells	Esharkawy et al. (2022)
28	Echinulin	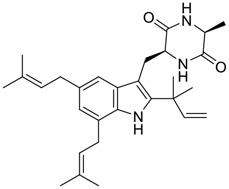	3.90	Vero E6 cells	Alhadrami et al. (2022)
29	EGCG	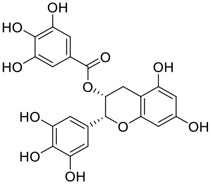	4.24	Vero E6 cells	Chiou et al. (2022)
30	Ellagic acid	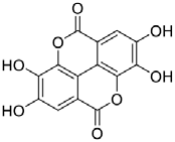	11.8	Vero E6 cells	Bahun et al. (2022)
31	(-)-Gallocatechin gallate	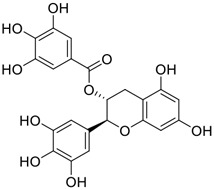	5.77	Vero E6 Cells	Xiao et al. (2021)
32	Glabridin	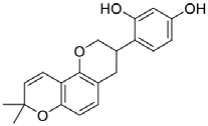	2.5	Vero E6 cells	Ngwe Tun et al. (2022)
33	Hesperidin	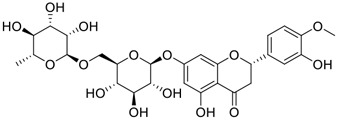	51.5	Vero E6 cells	Huang et al. (2022)
34	Isopetasin	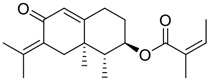	0.37	Vero E6 cells	Urda et al. (2022)
35	Ivermectin	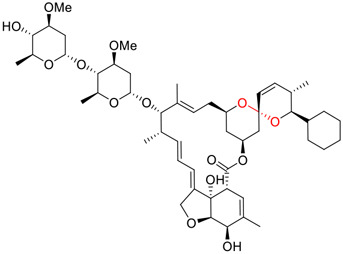	0.55	Vero E6 cells	Chable-Bessia et al. (2022)
36	Licoflavone C	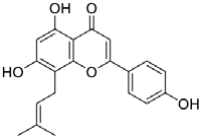	1.34	Vero E6 cells	Corona et al. (2022)
37	LPC (14:0/0:0)	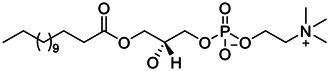	0.92	Vero E6 cells	Du et al. (2022)
38	LPC (16:0/0:0)	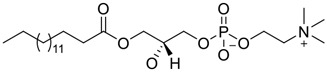	1.48	Vero E6 cells	Du et al. (2022)
39	LPC (16:0/18:1)	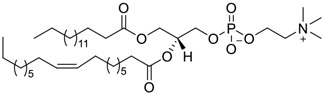	0.14	Vero E6 cells	Du et al. (2022)
40	Myricetin	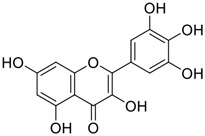	0.63	Vero E6 cells	Kato et al. (2021), Su et al. (2021)
41	Neferine	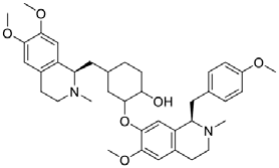	0.36	HEK293/hACE2 cells	Yang et al. (2021b)
42	(+)-Neoechinulin A	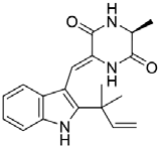	0.47	Vero E6 cells	Alhadrami et al. (2022)
43	Neoechinulin B	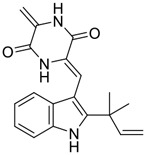	32.9	Vero E6 cells	Nishiuchi et al. (2022)
44	Neopetasin	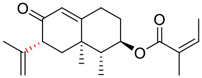	1.26	Vero E6 cells	Urda et al. (2022)
45	Oridonin	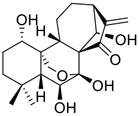	2.16	Vero E6 cells	Zhong et al. (2022)
46	Petasin	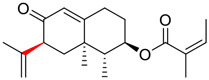	10.79	Vero E6 cells	Urda et al. (2022)
47	PGG	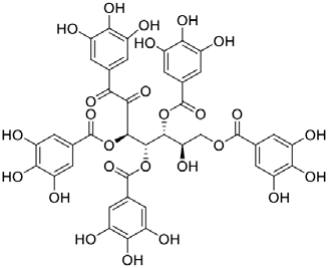	3.66	Vero E6 cells	Chiou et al. (2022)
48	Piniterpenoid A	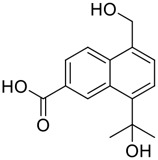	64.5	Vero E6 cells	Li et al. (2021b)
49	Piniterpenoid C	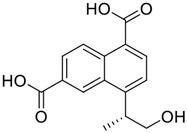	76.1	Vero E6 cells	Li et al. (2021b)
50	Plitidepsin	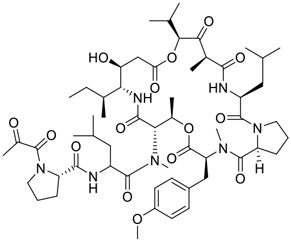	0.0043	Vero E6 cells	Guisado-Vasco et al. (2022), Sachse et al. (2022)
51	Punicalagin	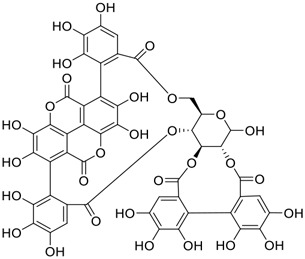	6.19	Vero E6 cells	Saadh et al. (2021) Suručić et al. (2021)
52	Resibufogenin	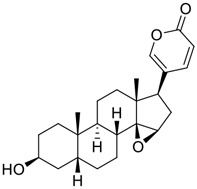	1.606	Vero E6 cells	Jin et al. (2021)
53	Salvianolic acid A	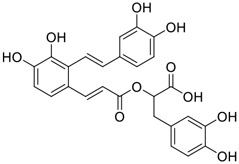	2.49	Vero E6 cells	Zhong et al. (2022)
54	Sangivamycin	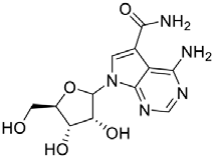	0.015	Vero E6 cells	Bennett et al. (2022)
55	Scutellarein	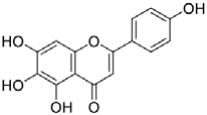	5.68	*E. coli* BL21 cells	Wu et al. (2022c)
56	(+)-Shikonin	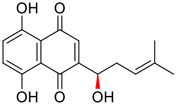	4.38	Vero E6 cells	Zhao et al. (2021), Ma et al. (2022)
57	Shikonin	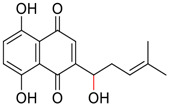	4.50	Vero E6 cells	Cui and Jia (2021), Zhao et al. (2021)
58	Sulforaphane	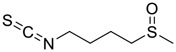	2.40	Caco-2 cells	Ordonez et al. (2022)
59	Tanshinone IIA	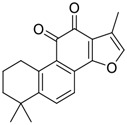	7.82 μg/mL	Vero E6 cells	Elebeedy et al. (2022)
60	Telocinobufagin	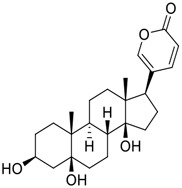	0.142	Vero E6 cells	Jin et al. (2021)
61	Thymohydroquinone	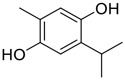	0.023 μg/mL	Vero E6 cells	Esharkawy et al. (2022)
62	Tubercidin	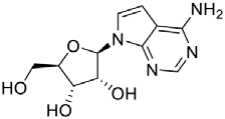	0.05	Calu-3 cells	Schultz et al. (2022)
63	Ugonin J	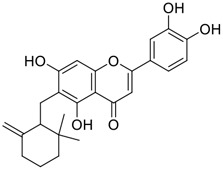	2.38	Vero E6 Cells	Chiou et al. (2021)
64	5,3′,4′-trihydroxyflavone	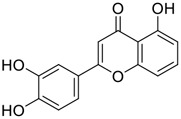	8.22	Vero E6 cells	Zhao et al. (2021)
65	7-OH-Cannabidiol	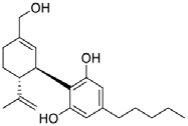	3.60	A549-ACE2 cells	Nguyen et al. (2022)

## 4 Conclusion and outlook

The devastating SARS-CoV-2 variants have caused over six million deaths worldwide. Natural products and small-molecule inhibitors have been widely studied (in *in vitro* studies, animal models, and clinical trials) and play an essential function in treating COVID-19. Drug research and development is a highly time‐consuming process. To date, Gilead’s controversial Veklury^®^ (Remdesivir, RdRp inhibitor) was conditionally approved to combat the outbreak. (Kalil et al., 2021; Wang and Yang, 2022a). Pfizer’s oral broad‐spectrum candidate Paxlovid^®^ (PF‐07321332, M^pro^ inhibitor) and Merck’s oral prodrug Lagevrio^®^ (Molnupiravir, RdRp inhibitor) raise new hope for a COVID‐19 cure. (Cully, 2022; Wang and Yang, 2022b). Promising clinical results have occurred, while small-molecule inhibitors still have a long way to go.

The substantial progress in treating COVID‐19 patients is not sufficient. Multiple factors must be considered. The first feasible factor, optimized drug combination therapy (such as gallinamide A + remdesivir, licorice-saponin A3 + PF‐07321332, telocinobufagin + molnupiravir, and cordycepin + tylophorine), targeting multiple targets, could not only enhance synergistic efficacy but also reduce drug resistance and toxicity. However, any potential combination would need to be tested *in vitro* and *in vivo* to verify the anticipated synergistic or additive effect. The second workable approach is natural product-based nanomedicines therapy. For example, the tylophorine-based lung-targeted liposome LP-NK007 could inhibit SARS-CoV-2 replication with a higher EC_50_ value *via* improving the accumulation and efficient delivery in the lung. Third, natural product-based lead optimization offers a valuable reference for enhancing anti‐SARS‐CoV‐2 potency and pharmacokinetic parameters. For example, taking gallinamide A as the lead, Payne et al. (Ashhurst et al., 2022) synthesized two highly selective SARS-CoV-2 cathepsin L inhibitors with nanomolar EC_50_ values. Taken together, we hope natural products (with the help of natural product-based nanomedicines therapy, lead optimization, and drug combination) prove to be a compelling direction in COVID-19 therapy.
